# Neurological Adverse Events Associated with the Use of Janus Kinase Inhibitors: A Pharmacovigilance Study Based on Vigibase

**DOI:** 10.3390/ph18030394

**Published:** 2025-03-11

**Authors:** Sunny Park, Min Kyu Kim, Sung Bin Park, Dong Hyeok Kim, Young Joo Byun, Soo An Choi

**Affiliations:** 1 Research Institute of Pharmaceutical Sciences, Korea University, Sejong 339-770, Republic of Korea; psunny0708@korea.ac.kr (S.P.);; 2 College of Pharmacy, Korea University, Sejong 339-770, Republic of Korea; rlaalsrb4564@korea.ac.kr (M.K.K.);

**Keywords:** JAK inhibitors, pharmacovigilance, neurological adverse events, memory impairment

## Abstract

**Background:** Janus kinase (JAK) inhibitors are a new class of targeted therapies that block cytokines and the signal transduction and activators of transcription (STAT) pathway. However, post-marketing surveillance studies have led to revised recommendations, highlighting potential serious heart-related events and cancer risk of JAK inhibitors. Here, we aimed to determine the neurological adverse events (AEs) of JAK inhibitors (tofacitinib, ruxolitinib, and baricitinib) based on a global real-world database. **Methods:** We analyzed individual case safety reports from the Uppsala Monitoring Center from January 1968 to 4 April 2022. A disproportionality analysis was performed using the proportional reporting ratio (PRR), reporting odds ratio (ROR), and information component (IC) to detect signals. Signals were classified according to the hierarchy of the Medical Dictionary for Regulatory Activities (MedDRA). Additionally, a stratified disproportionality analysis by age group and sex was performed for major AEs. **Results:** A total of 30,051,159 reports for all drugs were analyzed in this study. Among 105,798 reports of tofacitinib, 14.1% (14,863 reports) were neurological AEs. For ruxolitinib and baricitinib, 14.5% (6317 reports) and 10.2% (1216 reports) were neurological AEs, respectively. Various neurological AE signals were detected for tofacitinib and ruxolitinib, with memory impairment exhibiting the highest number of reports and a positive signal in the stratified disproportionality analysis by age group. Baricitinib did not reach the signal detection threshold. **Conclusions:** This study suggests the potential for neurological AEs, including memory impairment, associated with tofacitinib and ruxolitinib use based on a real-world database.

## 1. Introduction

Janus kinase (JAK) inhibitors represent a new class of targeted therapies with a potential immunomodulatory and anti-inflammatory effects that block cytokines and their signal transduction and activators of transcription (STAT) pathway, which are associated with various immune-related pathologies [1,2]. Over the past decade, the interest in targeted therapies within clinical immunology has grown. The introduction of JAK inhibitors has revolutionized the treatment landscape of various immunologic and hemato-oncologic diseases. Given the rapid development of these inhibitors, their safety data are crucial.

To date, JAK inhibitors have provided targeted oral therapies with efficacy comparable to tumor necrosis factor (TNF) inhibitors while maintaining a similar safety profile [3]. The results from the large post-marketing ORAL Surveillance study, which compared tofacitinib to TNF inhibitors, have led to altered recommendations for the use of JAK inhibitors [4]. Moreover, the U.S. Food and Drug Administration has recently added new black box warnings to all currently approved JAK inhibitors indicated for the treatment of arthritis and other inflammatory conditions [5]. These warnings highlight an increased risk of serious heart-related events, cancer, blood clots, and death [6].

Iatrogenic demyelination of the central nervous system (CNS) has been frequently reported in recent years owing to the expanded use of biologics [7]. Recent case reports have shown CNS demyelination in patients using tofacitinib, which could potentially be fatal, but the exact mechanism is unknown and requires further study [8,9]. Additionally, progressive multifocal leukoencephalopathy (PML) has been reported in a patient treated with ruxolitinib [10,11,12]. Neurological adverse effects, including dizziness, peripheral neuropathy, ataxia, aphasia, dysarthria, amnesia, and Wernicke encephalopathy, are known to be associated with JAK/STAT pathway inhibitors [13]. Neurological toxic effects led to the termination of the clinical development of the JAK1/JAK2 inhibitor and the consideration of the premature termination of fedratinib [14,15]. Despite these concerns, there has been limited focus on the safety issues related to JAK inhibitors, and additional research is lacking.

Evaluating safety after integrating post-marketing information is essential, particularly for new medications with uncertain safety profiles. VigiBase, the WHO’s global database, compiles, evaluates, and analyzes individual case safety reports (ICSRs) from spontaneous adverse event (AE) reports. These reports are crucial, with over 60% of the European Medicines Agency’s safety data from July 2012 to December 2013 coming from such reports [16]. Therefore, pharmacovigilance (PV) data offer valuable real-world insights into drug safety [17]. In this study, we aimed to investigate the neurological adverse events (AEs) associated with post-marketing JAK/STAT pathway inhibitors through global pharmacovigilance data mining to provide a reference for its clinical monitoring and risk identification.

## 2. Results

### 2.1. Demographic Characteristics of Safety Reports

The demographics of AE reports related to the three JAK inhibitors are presented in Table 1. A total of 30,051,159 reports for all drugs were analyzed in this study. Among 105,798 reports for tofacitinib, 14.1% (14,863 reports) were neurological AEs. For ruxolitinib and baricitinib, 14.5% (6317 reports) and 10.2% (1216 reports) were neurological AEs, respectively. The proportion of reports with unknown sex and age was higher for ruxolitinib than for tofacitinib or baricitinib. Tofacitinib and ruxolitinib were mostly reported in the Americas and were spontaneous report types, whereas baricitinib was predominantly reported in Europe, and about half of the reports were from studies. The proportion of serious cases was higher in ruxolitinib than in tofacitinib and baricitinib. The major notifiers were consumers and non-health professionals. The major indications were RA for tofacitinib and baricitinib and myelofibrosis for ruxolitinib.

### 2.2. Disproportionality Analysis and Neurological AE Signals

**Figure 1:**
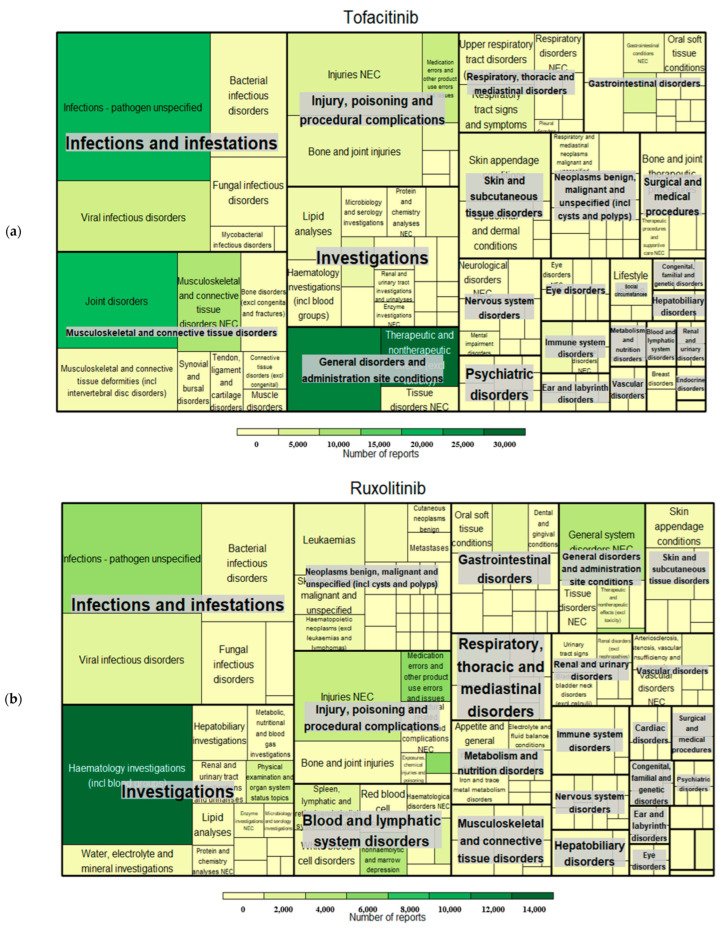

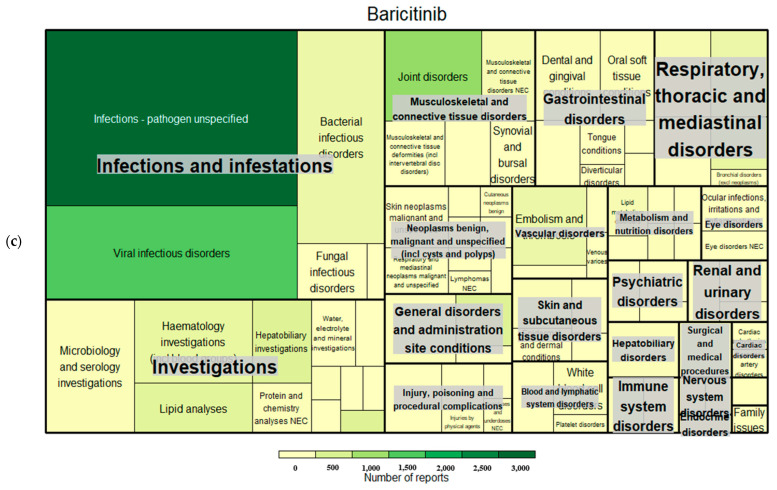
Treemaps of signals related to Janus kinase inhibitors: (**a**) tofacitinib; (**b**) ruxolitinib; and (**c**) baricitinib. Size: the number of signals. Color: the number of reports. NEC: not elsewhere classified.

The numbers of signals detected for tofacitinib, ruxolitinib, and baricitinib were 744, 951, and 296, respectively. Overall signal profiles are depicted as treemaps (Figure 1). The size represents the number of signals, and the color represents the number of reports. The most frequently reported and detected AEs as signals for the three JAK inhibitors were related to infections covering the largest area of the treemap.

Among the total positive signals, 24, 27, and 2 signals for tofacitinib, ruxolitinib, and baricitinib, respectively, were neurological AEs. Table 2 presents the disproportionality analysis results for neurological AE signals for the three JAK inhibitors.

While baricitinib showed neurological AE signals only for sciatica and post-herpetic neuralgia, various neurological AE signals were detected for tofacitinib and ruxolitinib. Memory impairment exhibited the highest number of reports among neurological AE signals for both tofacitinib and ruxolitinib, while baricitinib did not reach the signal detection threshold (n = 48; PRR: 1.13, ROR: 1.13, and IC_025_: −0.27). Tofacitinib showed a positive signal for memory impairment in the stratified analysis regardless of age and sex. A similar pattern was observed for ruxolitinib, although it was not positive for females. Table 3 shows the disproportionality analysis results for memory impairment stratified by age and sex.

## 3. Discussion

In this study, we determined the risk of neurological AEs associated with three JAK inhibitors based on a comprehensive global real-world database. Disproportionality analysis showed that tofacitinib and ruxolitinib exhibited various neurological AEs, while baricitinib showed only two neurological AE signals. Specifically, memory impairment was the most frequently reported neurological AE for tofacitinib and ruxolitinib, independent of age or sex. It seems that baricitinib has relatively low risk of neurological AEs; however, there is a need to collect more reports due to the relatively small number of reports.

Several confounding factors can affect the risk of neurological AEs. In particular, underlying diseases could affect AE occurrence. Rheumatoid arthritis, an indication for tofacitinib and baricitinib, has been studied for its association with an increased risk of developing dementia, possibly owing to reduced blood flow to vital organs [18]. Our study showed a positive signal for memory impairment with ruxolitinib, which is not indicated for rheumatoid arthritis. Despite the small number of reports, memory impairment was not detected as a signal in baricitinib, and about three fourths of baricitinib cases were for RA. In addition, the effect of underlying disease is complex and unstable over the course of treatment; for example, a study showed differences in the risk of dementia across different treatment groups [19]. This suggests that memory impairment should not be considered simply as a disease-related issue. Another important confounder on memory impairment is age. Given that memory loss is associated with aging [20], our finding of age-independent memory impairment signals suggests potential drug-related effects.

Tofacitinib exhibits specificity for JAK1/3 inhibitors, and ruxolitinib and baricitinib are JAK1 and JAK2 inhibitors, respectively [21,22]. Higher selectivity of JAK inhibitors is considered to decrease the off-target effect and be safe [21]; however, JAK selectivity is known to be relative, not absolute [23]. As the first-generation JAK inhibitors, these three JAK inhibitors showed low selectivity [24]. In addition, the mechanisms for safety are not fully understood, and it is not known whether JAK specificity alters the safety profile [2,25]. The results of this study, showing the various neurological AE signals in tofacitinib and ruxolitinib rather than baricitinib, could not be fully explained by JAK selectivity either.

The possibility of infection-related mechanisms cannot be ruled out when explaining neurological AEs. There are a few reports showing that neuropathy is related to tyrosine kinase inhibitors like imatinib [26,27] and dasatinib [28,29]. Similar to TNF-α inhibitors, which have been associated with neurological inflammation caused by infection [30,31,32], infections following JAK inhibitors are well-documented [33,34]. Our real-world database analysis also highlighted infections as major AEs associated with the three JAK inhibitors (Figure 1). PML, a demyelinating infection of the CNS caused by the John Cunningham (JC) polyomavirus [35], is associated with various neurological symptoms, including loss of coordination, motor weakness, mental impairment, and memory loss [36]. Given that PML cases have been reported after JAK inhibitor use [9,12,37] and memory impairment was mainly reported among neurological AEs in our study, vigilance regarding memory impairment related to JAK inhibitors is warranted.

Several studies, based on animal research, have suggested that the inhibition of the JAK2/STAT pathway contributes to memory impairment. The theory that amyloid β-dependent inactivation of the JAK/STAT axis in hippocampal neurons induces memory impairment via cholinergic dysfunction has been proposed [38]. Similarly, JAK/STAT signaling autoregulates the onset of astrogliogenesis, a process important for memory formation [39]. Additionally, the activation of JAK2/STAT3 signaling has been identified as a crucial component in a trimethyltin-induced cognitive dysfunction mouse model [40]. Interleukin (IL)-6, downregulated by tofacitinib [41], was considered to attenuate cognitive dysfunction in this model [40]. Furthermore, ruxolitinib has been identified as a potent inhibitor of Ca^2+^-calmodulin-dependent kinase II [42], which is essential for synaptic memory [43].

In addition to memory impairment, clinical cases reported peripheral neuropathy associated with JAK inhibitors. Peripheral neuropathy has been reported following tofacitinib use [44]. Momelotinib showed peripheral neuropathy in 44% of patients, and Wernicke encephalopathy was observed in seven patients treated with fedratinib during clinical trials [14]. Consistent with these findings, the present study highlights peripheral neuropathy as well as memory impairment and suggests the various neurological AEs associated with tofacitinib and ruxolitinib, thus highlighting the need for attention to neurological AEs of JAK inhibitors.

Conversely, several studies have implied that the inhibition of the JAK/STAT pathway could potentially improve nervous or cognitive impairment. For instance, the JAK2/STAT3 pathway has been shown to reduce amyloid β neurotoxicity by enhancing nicotinic acetylcholine receptors [45]. Another study demonstrated that the inhibition of STAT3 phosphorylation can attenuate learning and memory impairment in animal models of Alzheimer’s disease [46]. Recent research has suggested cognitive-improving actions of tofacitinib [47,48] and baricitinib [49,50]. Based on machine learning analyses, JAK inhibitors have been identified as promising candidates for drug repurposing in the treatment of Alzheimer’s disease [51]. Moreover, data from claims databases indicate that the incidence of dementia was lower in patients treated with biologics or targeted synthetic disease-modifying antirheumatic drugs (b/tsDMARDs) compared to those receiving conventional synthetic DMARDs [19]. This suggests that b/tsDMARDs may be associated with a reduced risk of dementia. Therefore, further evaluation is necessary to investigate neurological AEs, including memory impairment, associated with JAK inhibitors, especially in light of conflicting results in the literature.

Due to the association of JAK inhibitors with multiple different cytokines [52], several studies have been conducted to expand its therapeutic indications. Some studies of clinical trials for hospitalized COVID-19 patients showed little concern for neurological AEs in JAK inhibitors [53,54,55]. Meanwhile, the data source of AE reports we used did not have information on patients who did not experience AEs and had a lot of missing data or limited variables due to the retrospective data collection. Thus, confounder effects related to AE signals, including disease state, still exist because a control group adjusted for confounders is not available. However, clinical trials have a relatively short-term period of follow-up to observe the neurological AEs, including memory impairment. On the other hand, pharmacovigilance studies based on large databases have an advantage of being based on various patient populations and long-term observation periods compared to clinical trials. Although the potential of disease-related AEs cannot be ruled out due to lack of a control group without disease, our pharmacovigilance study provides the starting point for drug safety research with some advantages.

This study has several limitations. First, we could not account for JAK selectivity or blood–brain barrier penetration for the three JAK inhibitors. Second, most evidence to date regarding memory impairment related to JAK inhibitors stems from animal models, which present conflicting findings. Moreover, inherent limitations of spontaneous reporting databases, such as not being able to control confounders or reporting biases, constrain the robustness of our findings. Medical records including lab values cannot be obtained due to the characteristics of the database.

Nevertheless, pharmacovigilance activity based on large and long-term collected databases is valuable in that it provides a basis for further research. From July 2012 to December 2013, over 60% of safety information was obtained from spontaneous AE reports in the European Medicines Agency [16]. Our study underscores the potential for neurological AEs, including memory impairment, associated with the JAK inhibitors based on real-world clinical data. These findings highlight the need for further research to elucidate these drug-related effects and implications for clinical practice. As a starting point for further research, this study offers clues about further evaluation and suggests the enhanced monitoring of tofacitinib and ruxolitinib use, particularly neurological AEs.

## 4. Materials and Methods

### 4.1. Data Source

As of 28 November 2021, three JAK inhibitors (tofacitinib, ruxolitinib, and baricitinib) with more than 10,000 reports were selected for analysis. Individual case safety reports (ICSRs) from the World Health Organization Uppsala Monitoring Center (WHO-UMC), VigiBase^®^, were used in this study. VigiBase is the global database for spontaneous AE reporting, and this study used 30,051,159 AE reports covering the period from 1968 to 4 April 2022. The dataset included all suspected, interacting, and concomitant AE reports. The data fields included a unique number identifying each report, reported date, continent of the primary source, reporter, age, sex, drug name, indication, seriousness, and name of the AE as coded by the Medical Dictionary for Regulatory Activities (MedDRA) terminology. The seriousness of AEs was defined as resulting in death, life-threatening situations, persistent or significant disability or incapacity, hospital admission, or prolonged hospital stay [56]. ICSRs were reported by local physicians, pharmacists, other healthcare providers, and the public.

This study was approved by the Institutional Review Board of Korea University. The requirement for informed consent was waived owing to the use of secondary data (IRB No. 2020–0208). Data use was approved by the UMC, and the data are available at https://who-umc.org/ (request number ER096-2021) after payment. All procedures were performed in accordance with the relevant guidelines and regulations.

### 4.2. Data Mining and Signal Detection Criteria

To detect potential safety signals, we used disproportionality analysis, a method for identifying hidden patterns in a large database by comparing the proportion of occurring AEs between a specific drug and all other drugs. A signal is defined as reported information on a possible causal relationship between an AE and a drug by the WHO [57] and gives a new or known AE potentially caused by a drug, suggesting the need for further evaluation. For signal detection, a two-by-two contingency table was generated (Table 4).

The following three disproportionality parameters have been frequently used: the proportional reporting ratio (PRR), reporting odds ratio (ROR), and information component (IC) based on the Bayesian confidence propagation neural network [58,59,60]. The PRR is easy to calculate and interpret [58], and the ROR has been shown to have an advantage of correcting for bias due to the low number of reports compared to the PRR [59]. IC, based on a different methodology, is calculated by a logarithmic scale and by comparing the observed and expected reporting of a drug–AE combination [61]. For AEs reported at least three times, the calculation methods and signal detection criteria for these indices are listed in Table 5. For simplicity, signals were detected when the AEs of a drug of interest were reported more disproportionately than those of other drugs [62,63,64]. In this study, signals were considered to be detected when all criteria were met simultaneously. Additionally, a stratified disproportionality analysis by age group and sex was performed for major AEs. All statistical analyses were performed using the SAS statistical software (version 9.4, SAS Institute Inc., Cary, NC, USA) and Microsoft Excel (2021).

### 4.3. Hierarchical Analysis

The global standard terminology for recording AEs and medical histories (MedDRA) was used [65]. It has a five-level hierarchy: system organ class (SOC), high-level group term (HLGT), high-level term (HLT), preferred term (PT), and lowest-level term (LLT) [66]. Disproportionality analysis was performed at the PT level, and hierarchical analysis was conducted to identify neurological AE signals using MedDRA version 26.1. Neurological AEs were defined as PTs belonging to the nervous system disorder SOC. The positive signals were graphically visualized using a treemap package in R Studio version 4.1.2. Treemaps are a data visualization technique for hierarchical datasets and capture the value of individual data points and the structure of the hierarchy [67]. The treemap was depicted using different sizes and colors, representing the number of signals and reports, respectively.

## 5. Conclusions

This study suggests the potential for neurological AEs, including memory impairment, associated with tofacitinib and ruxolitinib use based on a real-world database. The identification of potential neurological AEs provides valuable insights for clinicians and regulatory authorities.

## Figures and Tables

**Table 1:** Demographics of the total and neurological AE reports of tofacitinib, ruxolitinib, and baricitinib.

	Tofacitinib		Ruxolitinib		Baricitinib	
	Total Reports (*N* = 105,798)	Neurological AE* Reports (*N* = 14,863)	Total Reports (*N* = 43,661)	Neurological AE* Reports (*N* = 6317)	Total Reports (*N* = 11,963)	Neurological AE* Reports (*N* = 1216)
Sex						
Male	19,933 (18.8%)	2297 (15.5%)	11,856 (27.2%)	1366 (21.6%)	2399 (20.1%)	182 (15.0%)
Female	82,018 (77.5%)	12,292 (82.7%)	10,517 (24.1%)	1675 (26.5%)	9171 (76.7%)	1009 (83.0%)
Unknown	3847 (3.7%)	271 (1.8%)	21,288 (48.7%)	3276 (51.9%)	393 (3.2%)	25 (2.0%)
Age						
<18 years	388 (0.4%)	32 (0.2%)	344 (0.8%)	21 (0.3%)	42 (0.5%)	1 (0.1%)
18–44 years	10,326 (9.8%)	1437 (9.7%)	746 (1.7%)	102 (1.6%)	930 (7.7%)	96 (7.9%)
45–64 years	43,264 (40.9%)	6810 (45.8%)	4216 (9.7%)	644 (10.2%)	3581 (29.9%)	417 (34.3%)
65–74 years	21,802 (20.6%)	3202 (21.6%)	5113 (11.7%)	725 (11.5%)	1946 (16.3%)	199 (16.4%)
≥75 years	10,299 (9.7%)	1565 (10.5%)	4670 (10.7%)	694 (11.0%)	1022 (8.5%)	105 (8.6%)
Unknown	19,719 (18.6%)	1817 (12.2%)	28,572 (65.4%)	4131 (65.4%)	4442 (37.1%)	398 (32.7%)
Serious						
Yes	33,052 (31.2%)	5865 (38.2%)	21,573 (49.4%)	3023 (47.8%)	3285 (27.5%)	392 (32.3%)
No	72,405 (68.4%)	9137 (61.5%)	21,497 (49.2%)	3225 (51.1%)	8673 (72.5%)	823 (67.7%)
Unknown	341 (0.4%)	41 (0.3%)	591 (1.4%)	69 (1.1%)	5 (0.0%)	1 (0.1%)
Notifier						
Physician	24,507 (23.2%)	2613 (17.6%)	12,668 (29.0%)	1583 (25.1%)	2900 (24.2%)	231 (19.0%)
Pharmacist	3639 (3.4%)	606 (4.1%)	1810 (4.2%)	294 (4.6%)	800 (7.4%)	59 (4.8%)
Other Health Professional	22,534 (21.3%)	3357 (22.6%)	5370 (12.3%)	697 (11.0%)	824 (6.9%)	70 (5.8%)
Consumer/Non-Health Professional	53,971 (51.0%)	8162 (54.9%)	22,904 (52.5%)	3675 (58.2%)	7211 (60.3%)	844 (69.4%)
Lawyer	66 (0.1%)	8 (0.1%)	19 (0.0%)	5 (0.1%)	1 (0.0%)	1 (0.1%)
Unknown	1081 (1.0%)	117 (0.8%)	890 (2.0%)	63 (1.0%)	147 (1.2%)	11 (0.9%)
UN continent						
Americas	97,351 (92.0%)	14,046 (94.5%)	33,382 (76.5%)	5375 (85.1%)	1780 (14.9%)	150 (12.3%)
Europe	5700 (5.4%)	613 (4.1%)	6614 (15.1%)	766 (12.1%)	9455 (79.0%)	1004 (82.6%)
Asia	2155 (2.1%)	126 (0.9%)	3284 (7.5%)	160 (2.5%)	624 (5.2%)	48 (4.0%)
Oceania	559 (0.5%)	76 (0.5%)	348 (0.8%)	14 (0.2%)	67 (0.6%)	9 (0.7%)
Africa	33 (0.0%)	2 (0.0%)	33 (0.1%)	2 (0.0%)	37 (0.3%)	5 (0.4%)
First Date Database						
2010~2014	2323 (2.2%)	394 (2.6%)	3127 (7.2%)	442 (7.0%)	5 (0.0%)	0 (0.0%)
2015	5093 (4.8%)	916 (6.2%)	3345 (7.6%)	485 (7.7%)	4 (0.0%)	0 (0.0%)
2016	5769 (5.5%)	939 (6.3%)	3366 (7.7%)	525 (8.3%)	5 (0.0%)	1 (0.1%)
2017	11,717 (11.1%)	1940 (13.0%)	5786 (13.3%)	1014 (16.1%)	91 (0.8%)	13 (1.1%)
2018	20,083 (19.0%)	2495 (16.8%)	7154 (16.4%)	1156 (18.3%)	1369 (11.4%)	161 (13.2%)
2019	25,297 (23.9%)	3191 (21.5%)	7793 (17.8%)	1180 (18.7%)	3411 (28.5%)	357 (29.4%)
2020	17,261 (16.3%)	2447 (16.5%)	5883 (13.5%)	696 (11.0%)	3001 (25.1%)	384 (23.3%)
2021	17,293 (16.3%)	2470 (16.6%)	6978 (16.0%)	804 (12.7%)	3465 (29.0%)	353 (29.0%)
2022.4	962 (0.9%)	71 (0.5%)	229 (0.5%)	15 (0.2%)	612 (5.1%)	47 (3.9%)
Report Type						
Spontaneous	101,750 (96.2%)	14,443 (97.2%)	31,923 (73.1%)	4647 (73.6%)	5501 (46.0%)	520 (42.8%)
From study	3650 (3.5%)	375 (2.5%)	11,175 (25.6%)	1592 (25.2%)	6429 (53.7%)	520 (56.7%)
Indication ^§^						
Rheumatoid Arthritis	55,028 (52.0%)	8828 (59.4%)	3 (0.0%)	2 (0.0%)	8661 (72.4%)	920 (75.7%)
Other ^§§^	9462 (8.9%)	1228 (8.3%)	35,601 (81.6%)	5560 (88.0%)	1211 (10.1%)	100 (8.2%)
Missing	41,308 (39.0%)	4807 (32.3%)	8007 (18.4%)	755 (12.0%)	2091 (17.5%)	196 (16.1%)

^§^ One case reported one or more indications. ^§§^ Other cases included those without rheumatoid arthritis-related indications. The most frequently reported indications were psoriatic arthropathy and ulcerative colitis for tofacitinib, myelofibrosis and polycythaemia vera for ruxolitinib, and COVID-19 and atopic dermatitis for baricitinib. * AE: adverse event.

**Table 2:** Disproportionality analysis for neurological AEs of JAK inhibitors.

Drugs	HLGT *	PT *	Number of Reports	PRR * (95% CI *)	ROR * (95% CI *)	IC * (95% CI *)
Tofacitinib	Mental impairment disorders	Memory impairment	1275	3.42 (3.23–3.61)	3.44 (3.26–3.64)	1.76 (1.68–1.84)
		Dementia	159	2.63 (2.25–3.07)	2.63 (2.25–3.08)	1.38 (1.15–1.60)
		Dementia Alzheimer’s type	50	2.88 (2.18–3.80)	2.88 (2.18–3.80)	1.49 (1.06–1.86)
	Neurological disorders NEC	Dysstasia	232	2.56 (2.25–2.91)	2.56 (2.25–2.92)	1.34 (1.15–1.52)
		Dysgraphia	80	4.54 (3.64–5.67)	4.55 (3.65–5.67)	2.14 (1.80–2.43)
		Cerebral disorder	40	2.68 (1.97–3.66)	2.68 (1.97–3.66)	1.39 (0.90–1.80)
		Post-herpetic neuralgia	35	4.31 (3.09–6.02)	4.32 (3.09–6.03)	2.03 (1.51–2.47)
		Dyslexia	18	6.53 (4.09–10.42)	6.53 (4.09–10.42)	2.48 (1.74–3.07)
		Tinel’s sign	3	23.59 (7.26–76.59)	23.59 (7.26–76.60)	2.46 (0.41–3.65)
	Movement disorders (incl. parkinsonism)	Movement disorder	315	3.07 (2.75–3.43)	3.08 (2.75–3.44)	1.60 (1.44–1.76)
		Fine motor skill dysfunction	28	3.75 (2.58–5.44)	3.75 (2.5–5.44)	1.82 (1.24–2.31)
		Bradykinesia	27	2.01 (1.37–2.93)	2.01 (1.37–2.93)	0.97 (0.38–1.47)
		Essential tremor	8	2.69 (1.34–5.40)	2.69 (1.34–5.40)	1.28 (0.11–2.11)
	Peripheral neuropathies	Carpal tunnel syndrome	179	5.15 (4.45–5.97)	5.16 (4.45–5.98)	2.33 (2.11–2.53)
		Nerve compression	134	6.21 (5.24–7.37)	6.22 (5.24–7.38)	2.58 (2.33–2.82)
		Sciatic nerve neuropathy	10	10.14 (5.40–19.06)	10.15 (5.40–19.07)	2.79 (1.76–3.55)
	Spinal cord and nerve root disorders	Sciatica	172	4.42 (3.80–5.14)	4.42 (3.81–5.14)	2.11 (1.89–2.32)
		Spinal cord compression	18	2.45 (1.54–3.90)	2.45 (1.54–3.90)	1.23 (0.49–1.82)
		Spinal cord disorder	9	2.32 (1.21–4.48)	2.32 (1.21–4.48)	1.11 (0.02–1.90)
		Radicular pain	9	8.14 (4.20–15.79)	8.14 (4.20–15.79)	2.54 (1.45–3.33)
	Headaches	Sinus headache	71	2.77 (2.19–3.50)	2.77 (2.20–3.50)	1.44 (1.09–1.76)
		Cold-stimulus headache	6	4.17 (1.86–9.34)	4.17 (1.86–9.34)	1.73 (0.36–2.66)
	Central nervous system vascular disorders	Intracranial aneurysm	21	2.10 (1.36–3.22)	2.10 (1.36–3.22)	1.03 (0.34–1.58)
	Neuromuscular disorders	Amyotrophic lateral sclerosis	12	2.51 (1.42–4.43)	2.51 (1.42–4.43)	1.24 (0.31–1.94)
Ruxolitinib	Mental impairment disorders	Memory impairment	696	4.50 (4.18–4.85)	4.56 (4.23–4.91)	2.16 (2.05–2.26)
		Dementia	67	2.68 (2.11–3.40)	2.68 (2.11–3.41)	1.40 (1.03–1.72)
	Sleep disturbances (incl. subtypes)	Hypersomnia	132	3.15 (2.65–3.73)	3.15 (2.66–3.74)	1.64 (1.38–1.87)
	Neurological disorders NEC	Postural dizziness	54	2.77 (2.12–3.62)	2.77 (2.12–3.62)	1.44 (1.03–1.80)
		Post-herpetic neuralgia	36	10.78 (7.76–14.98)	10.79 (7.76–15.00)	3.23 (2.72–3.66)
	Spinal cord and nerve root disorders	Sciatica	60	3.71 (2.88–4.77)	3.71 (2.88–4.78)	1.85 (1.46–2.19)
		Lumbar radiculopathy	5	3.70 (1.54–8.92)	3.70 (1.54–8.92)	1.57 (0.04–2.56)
	Peripheral neuropathies	Polyneuropathy	34	2.34 (1.67–3.28)	2.34 (1.67–3.28)	1.20 (0.67–1.64)
		Nerve compression	21	2.32 (1.51–3.56)	2.32 (1.51–3.56)	1.17 (0.49–1.72)
		Axonal neuropathy	5	5.78 (2.40–13.93)	5.78 (2.40–13.93)	2.00 (0.48–3.00)
	Neuromuscular disorders	Neuromuscular blockade	22	10.78 (7.08–16.43)	10.79 (7.08–16.44)	3.13 (2.46–3.67)
	Encephalopathies	Leukoencephalopathy	8	3.02 (1.51–6.04)	3.02 (1.51–6.04)	1.43 (0.26–2.26)
		Metabolic encephalopathy	8	3.02 (1.51–6.05)	3.02 (1.51–6.05)	1.43 (0.26–2.26)
	Movement disorders (incl. parkinsonism)	Clumsiness	14	3.69 (2.18–6.23)	3.69 (2.18–6.24)	1.75 (0.90–2.41)
	Structural brain disorders	Cerebral atrophy	10	2.91 (1.56–5.42)	2.91 (1.56–5.42)	1.41 (0.38–2.17)
	Cranial nerve disorders (excl. neoplasms)	Vocal cord paralysis	8	2.76 (1.38–5.52)	2.76 (1.38–5.52)	1.32 (0.15–2.15)
	Central nervous system vascular disorders	Carotid arteriosclerosis	4	4.93 (1.84–13.17)	4.93 (1.84–13.17)	1.77 (0.04–2.85)
Baricitinib	Spinal cord and nerve root disorders	Sciatica	13	2.92 (1.70–5.03)	2.92 (1.70–5.04)	1.45 (0.56–2.12)
	Neurological disorders NEC *	Post-herpetic neuralgia	10	10.82 (5.81–20.13)	10.83 (5.82–20.15)	2.88 (1.85–3.63)

* PRR, proportional reporting ratio; ROR, reporting odds ratio; IC, information component; CI, confidence interval; PT, preferred term; HLGT, high-level group term; NEC, not elsewhere classified; incl., including; excl., excluding.

**Table 3:** Disproportionality analysis for memory impairment stratified by age and sex.

Drugs	Stratum		Number of Reports	PRR * (95% CI *)	ROR * (95% CI *)	IC * (95% CI *)
Tofacitinib	**All ^§^**		**1275**	3.42 (3.23–3.61)	3.44 (3.26–3.64)	1.76 (1.68–1.84)
	Age group	12–17 years	1	1.63 (0.23–11.54)	1.63 (0.23–11.64)	0.43 (−3.37–2.07)
		**18**–**44 years ^§^**	**131**	4.68 (3.94–5.55)	4.73 (3.98–5.62)	2.20 (1.94–2.43)
		**45**–**64 years ^§^**	**556**	3.45 (3.18–3.75)	3.48 (3.20–3.79)	1.76 (1.64–1.88)
		**65**–**74 years ^§^**	**347**	4.94 (4.44–5.49)	5.00 (4.49–5.57)	2.26 (2.10–2.41)
		**≥75 years ^§^**	**191**	5.55 (4.81–6.39)	5.63 (4.87–6.51)	2.43 (2.22–2.63)
	Sex	**Male ^§^**	**204**	3.43 (2.99–3.93)	3.45 (3.01–3.96)	1.76 (1.56–1.95)
		**Female ^§^**	**1110**	3.15 (2.97–3.34)	3.18 (3.20–3.79)	1.64 (1.55–1.72)
Ruxolitinib	**All ^§^**		**696**	4.50 (4.18–1.85)	4.56 (4.23–4.91)	2.16 (2.05–2.26)
	Age group	18–44 years	3	1.38 (0.45–4.26)	1.38 (0.44–4.28)	0.39 (−1.66–1.58)
		**45**–**64 years ^§^**	**45**	2.81 (2.10–3.76)	2.83 (2.11–3.79)	1.46 (1.01–1.85)
		**65**–**74 years ^§^**	**35**	2.04 (1.47–2.84)	2.05 (1.47–2.86)	1.01 (0.49–1.44)
		**≥ 75 years ^§^**	**39**	2.45 (1.79–3.35)	2.46 (1.79–3.37)	1.26 (0.77–1.68)
	Sex	Male	61	1.71 (1.33–2.19)	1.71 (1.33–2.20)	0.76 (0.38–1.10)
		**Female ^§^**	**108**	2.36 (1.95–2.85)	2.37 (1.96–2.87)	1.23 (0.94–1.49)
Baricitinib	All		48	1.13 (0.85–1.50)	1.13 (0.85–1.50)	0.17 (−0.27–0.55)
	Age group	18–44 years	5	1.89 (0.79–4.53)	1.89 (0.79–4.56)	0.81 (−0.72–1.80)
		45–64 years	19	1.40 (0.89–2.19)	1.40 (0.89–2.20)	0.47 (−0.25–1.04)
		65–74 years	6	0.93 (0.42–2.07)	0.93 (0.42–2.07)	−0.10 (−1.47–0.83)
		≥ 75 years	3	0.86 (0.28–2.66)	0.86 (0.28–2.67)	−0.19 (−2.24–1.00)
	Sex	Male	4	0.54 (0.20–1.43)	0.54 (0.20–1.43)	−0.82 (−2.55–0.26)
		Female	44	1.12 (0.84–1.51)	1.12 (0.84–1.51)	0.17 (−0.29–0.56)

^§^ Signals; * PRR, proportional reporting ratio; ROR, reporting odds ratio; IC, information component; CI, confidence interval.

**Table 4:** Two-by-two contingency table for disproportionality analysis.

Number of Reports	AE*s of Interest	All Other AE*s
Drug of interest	A	B
All other drugs	C	D

The number of reports included in A: both target drugs and specific AEs; B: target drug AEs but with all other AEs; C: specific AEs but with all other drugs; and D: all other drugs and all other AEs. * AE: adverse event.

**Table 5:** Formulae and criteria for signal detection.

Indices	Formula	Positive Signal Criteria
PRR *	[A/(A+B)]/[C/(C+D)]	PRR ≥ 2
ROR *	(A/B)/(C/D)	ROR ≥ 2
IC *	IC = log_2_P*(AE, Drug)/P(AE)P(Drug)	Lower limit of 95% CI *≥ 0

* PRR, proportional reporting ratio; ROR, reporting odds ratio; IC, information component; P, probability; AE, adverse event; CI, confidence interval.

## Data Availability

The datasets are not publicly available due to the ongoing collection of AE reports. However, they are available from UMC upon reasonable request. The transfer, rent, or sale of the database to any third party other than researchers is prohibited. Data will be available after obtaining approval from the UMC at http://who-umc.org/ (request number ER096-2021).

## Abbreviations

The following abbreviations are used in this manuscript:

JAK	Janus kinase
STAT	Signal transduction and activators of transcription
AE	Adverse event
PRR	Proportional reporting ratio
ROR	Reporting odds ratio
IC	Information component
MedDRA	Medical dictionary for regulatory activities
SOC	System organ class
HLGT	High-level group term
HLT	High-level term
PT	Preferred term
LLT	Lowest-level term
TNF	Tumor necrosis factor
CNS	Central nervous system
PML	Progressive multifocal leukoencephalopathy
ICSRs	Individual case safety reports
PV	Pharmacovigilance
CI	Confidence interval
NEC	Not elsewhere classified
